# Eugenics and the Genetics Society

**DOI:** 10.1038/s41437-026-00838-5

**Published:** 2026-04-20

**Authors:** Alex Aylward, Daniel J. Fairbanks, Maria Kiladi, Gregory Radick

**Affiliations:** 1 Alex Aylward, Faculty of History, University of Oxford, Stephen A. Schwarzman Centre for the Humanities, Radcliffe Observatory Quarter, Oxford, UK; 2 Daniel J. Fairbanks, Department of Biology, Utah Valley University, Orem, UT USA; 3 Maria Kiladi, Department of Political Science, University College London, London, UK; 4 Gregory Radick, School of Philosophy, Religion and History of Science, University of Leeds, Leeds, UK

**Keywords:** Genetic counselling, Genetic variation

## Abstract

Genetics and eugenics co-evolved at the beginning of the twentieth century and remained associated through the 1940s and beyond. Early geneticists were far from unanimous in their views on eugenics; some avidly supported the movement, whereas others openly opposed it or chose to remain detached. Academic institutions and scientific societies are currently reckoning with their past associations with eugenics. This article highlights historical connections between the UK Genetics Society and the eugenics movement in Britain. The complexity of these connections is illustrated through case studies of three notable Genetics Society past Presidents—J. B. S. Haldane, R. A. Fisher, and L. S. Penrose—who represent diverse ways that British geneticists engaged with the eugenics movement, from career-long support to science-based opposition. Their contributions to, and critiques of, eugenics are situated in their scientific and historical contexts. We reflect on the historical role of professional genetics organisations in facilitating the rise of eugenics in the early twentieth century, and the present responsibility of the same organisations to combat its contemporary revival.

## Introduction

In recent years, a number of universities and learned societies have begun to reckon publicly with their roles in establishing and perpetuating eugenics (see, for example, American Society of Human Genetics 2023). The headline-making results so far have included the removal of memorials and the de-naming of lectures, prizes and spaces commemorating prominent eugenicists. Alongside these symbolic actions, careful historical scholarship on the past links of these bodies to eugenics is essential. In this spirit, the Executive Committee of the United Kingdom’s principal professional organisation for geneticists, the Genetics Society, and the editors of the Society’s journal, *Heredity*, approached us—a team of professional historians of science and a practising geneticist with deep historical interests—in February 2021 about offering an independent view of the connections between the Genetics Society and the eugenics movement, concentrating especially on past Presidents of the Society with ties to eugenics. Although very much a part of the wider institutional reckoning, the invitation was a direct response to controversy stirred by an article in *Heredity* decried by critics as a defence of the eugenical work of one such figure, the prominent statistician and geneticist R. A. Fisher (Bodmer et al. 2021). We gladly accepted in hopes of stimulating serious conversation and reflection on some of the more troubling aspects of the field’s history.

What follows is in no way intended to be comprehensive, and certainly not the final word on the matter. Much more modestly, we aim to convey something of the extent and nature of the Genetics Society’s multiple and multifaceted interactions with eugenics. The story is far from straightforward, for neither the Genetics Society (known as the Genetical Society until 2002) nor the eugenics movement represented a united front, and their boundaries were blurred significantly. By way of bringing out general features of that story without undue flattening of biographical particulars, we focus our attention on three of the Society’s past Presidents, examining their stated attitudes towards eugenics and their activities in either promoting or challenging the movement. Our choices—J. B. S. Haldane (President, 1932–36), Fisher (1940–43), and Lionel Penrose (1955–58)—are deliberate. Although all were white, Oxbridge-educated men, they illustrate not only the diversity of ways that geneticists in Britain engaged with the eugenics movement during and after its heyday, from ardent support to sharp critique, but also the importance that differences in how, when and where they first learned genetics could make to later judgements about hereditarian genetics and its eugenic ambitions. Their collective presidential tenures—ranging from the early 1930s to the late 1950s—span a period which saw both the growth of genetics as a well-established professional discipline and the erosion of previously widespread support for the organised eugenics movement. The cases of Haldane, Fisher and Penrose thus provide illuminating snapshots of this critical period, during which the relationship between genetics and eugenics was contested and ultimately transformed.

## A quarter century of entanglements, 1907–1932

To set the scene we begin with a brief survey of developments before any of these presidencies, taking as our starting point not the coining of ‘eugenics’ in 1883 by the London polymath and pioneering heredity researcher Francis Galton, nor the rediscovery of the Brünn Augustinian Gregor Mendel’s experimental study of plant hybrids in 1900, but the establishment of the Eugenics Education Society (EES) in London in 1907. First and foremost a propaganda outfit—‘Education’ was dropped from the name only in the mid-1920s—the EES’s guiding purpose in its early years was to disseminate eugenic knowledge and encourage eugenic sensibilities throughout the population, chiefly via lectures and publications (Soloway 1998, p 55). It also endeavoured to influence government policy in the direction of what was called ‘racial betterment’, or the improvement of the biological ‘quality’ of the national ‘stock’. Minor successes in the early days included the passage of the Mental Deficiency Act (1913)—legislation allowing individuals deemed ‘feeble-minded’ to be indefinitely institutionalised (though not sterilised, as was the wish of some eugenic lobbyists)—and the extension of Income Tax rebates to parents, so that having children would not financially disadvantage those in the ‘eugenically desirable’ middle and professional classes (Schenk and Parkes 1968).

By the outbreak of the Great War, EES membership—which included many medical professionals, politicians, lawyers, teachers, clergy, and a significant number of women—had grown to more than 1000 (MacKenzie 1976). But strength did not reside in numbers alone. To the extent that their propaganda rested on the latest scientific knowledge of inheritance, the EES’s leadership deemed it crucial to secure the public backing of the growing cadre of professional scientific experts in heredity. At the time, tensions simmered between the statistically focused followers of Galton (the so-called ‘Biometricians’) and the experimentally focused followers of Mendel (then ‘Mendelians’, later ‘geneticists’). In 1910, the EES’s President, the barrister Montague Crackanthorpe, used his Presidential Address to play peacemaker, positioning race betterment as a unifying purpose for all students of inheritance. ‘It would be a thousand pities if the Mendelians were to say to the Biometricians, “We have no need of you’’, said Crackanthorpe, ‘or if the Biometricians were to say to the Mendelians, “We have no need of you”, for Eugenics requires the services of both, as a little reflection will show’ (quoted in Mazumdar 1992, p 61). The next year, in a paper on ‘Heredity’ read at the second undergraduate meeting of the new Cambridge University Eugenics Society, the young Fisher—still an undergraduate himself, and a founder member (Mazumdar 1992, p 232)—seemingly answered that call, affirming the vision of a unified science in which eugenics, biometry and Mendelism walked together (‘I have’, declared Fisher, ‘almost entirely devoted myself to the two lines of modern research which are of particular interest in Eugenics, that is to Biometrics and Mendelism’) (Fisher 1911, p 51). But it was the formation of the Genetical Society in June 1919—envisaged as a learned society dedicated to fostering first-class research in the fledgling science of genetics—that offered the most tantalising prospects for institutional as well as intellectual harmonisation. From the start, the EES ‘desired to be on friendly terms’ with the new Society (Hodson 1924), inviting William Bateson, the Genetical Society’s co-founder (with Edith Rebecca Saunders), to deliver the EES’s annual Galton Lecture for 1919.

By then based at the John Innes Horticultural Institution in greater London, Bateson from 1900 onwards had been the Mendelian-in-chief: the man who had not only dubbed the new science ‘genetics’ (in 1905) but, in collaboration initially with Saunders and then with a growing number of their students, had made Cambridge University into genetics’ first world headquarters (Richmond 2001). That Bateson accepted the EES’s invitation (eventually delivering the Galton Lecture in 1921) was in keeping with his general sympathy for the aims of the organisation. Notwithstanding a still-lively historiographical tradition of downplaying the role of Mendelians in promoting hereditarian eugenics (see, e.g., Bodmer and Charlesworth 2025), Bateson routinely and approvingly acknowledged the eugenic bearing of genetic knowledge. In 1909 he published the seminal book *Mendel’s Principles of Heredity*, which concluded with a three-page section on ‘Sociological Applications’ wherein he envisaged the enactment of twentieth-century eugenic practice: ‘there can be little doubt that before long we shall find that communities more fully emancipated from tradition will make a practical application of genetic principles to their own population’ (Bateson 1909, p 304).

Nor was Bateson the first Cambridge geneticist to end a book with a section on eugenics. His junior ally Reginald Punnett had done the same in his short but influential book *Mendelism* (Punnett 1905), characterised as ‘the first genetics textbook’ (Edwards 2012). Appealing to those of his readers ‘who are troubled with the suspicion that hygiene and education are fleeting palliatives at best, which, in postponing, but augment the difficulties they profess to solve’, Punnett concluded the book with the sort of hereditarian absolutism that pervaded the eugenics movement in the coming decades:

To them the facts of heredity may speak with no uncertain voice. Education is to man what manure is to the pea. The educated are in themselves the better for it, but their experience will alter not one jot the irrevocable nature of their offspring. Permanent progress is a question of breeding rather than of pedagogics; a matter of gametes, not of training. As our knowledge of heredity clears and the mists of superstition are dispelled, there grows upon us with an ever increasing and relentless force the conviction that the creature is not made but born. (Punnett 1905, pp 59–60)

Such words reflect a clear assertion of heredity over environment as the source of phenotypic variation for intellectual ability (or, more direly, disability), and therefore a purported justification for eugenic intervention. Punnett reasserted those links again in the summer of 1912 in London, as a speaker at the EES-organised First International Eugenics Congress, whose delegates were welcomed by no less than the Rt. Hon. Arthur Balfour, the former UK Prime Minister and eponym of a recently established Cambridge chair, the Arthur Balfour Professorship of Genetics, held from that year by Punnett. According to the *British Medical Journal*, at the Congress Balfour stressed the ‘duty to convince the public that the study of eugenics was one of the greatest and most pressing necessities of our day’, with Punnett following up the next afternoon on ‘feeble-mindedness’—inherited in strict Mendelian fashion, Punnett claimed—as a promising target for elimination through segregation (British Medical Journal 1912).

The double act of Balfour and Punnett subsequently continued at the highest level of the Genetical Society, with Balfour serving as the first President, from 1919 to 1930, succeeded by Punnett from 1930 to 1932. Both men also strengthened their personal ties to the EES, Punnett joining up in 1912, and Balfour elected an honorary member the following year (‘List of Members A–Z’ 2012). Bateson, however, was generally far from keen on close integration between pure-science and applied-science organisations. In his view, as set out in a 1911 address, a well-functioning scientific system should encourage the advance of knowledge for knowledge’s sake, with no pressure to demonstrate the applied-science value of a scientific question or its answer (Bateson 1911; Radick 2013, pp 283–284). For sure, that system should also make room for specialists whose business is the application of the new knowledge for solving real-world problems—and again, Bateson regarded eugenics along with agriculture as one of the major applied-science wings of genetics (Mazumdar 1992, p 232; see also Radick 2023, pp 10‒11, 232‒233, 291, 480n72). But for there to be genuinely useful knowledge to apply in the first place, the pure scientists had to be left alone.

Accordingly, at the EES’s 1921 meeting, Bateson began his Galton Lecture by noting that he had ‘never seen my way to take a definite part in [the EES’s] activities nor even to become a member of your body’. Principally, he went on, this was due to his conviction that ‘the eugenist and the geneticist will … work most effectively without organic connexion, and though we have much in common we should not be brigaded together’. While eugenics—quite nobly, Bateson stressed—strove for the ‘betterment of the human race’, genetics, by contrast, was concerned with ‘a problem of pure physiology’. Too close an association between the two risked obscuring the ‘distinctness of our aims’. We see here Bateson’s concern with preserving the autonomy and credibility of the still fledgling science which he had helped invent. ‘Alliances between pure and applied science’, he continued, ‘are as dangerous as those of spiders, in which the fertilising partner is apt to be absorbed’ (all quotations from Bateson 1921, p 325).

Bateson’s death in February 1926 revived EES hopes for closer relations. That year Major Leonard Darwin, son of Charles Darwin and now President of the Eugenics Society (as it was renamed), began drafting a bill proposing to legalise the sterilisation, on eugenic grounds, of individuals deemed ‘mentally unfit’. In campaigning to have this bill pass through parliament, the Eugenics Society sought the public backing of recognised geneticists who could vouch for the policy’s sound basis in genetic science (Barker 1989; Macnicol 1989; Aylward and McGuire 2025). And so, in February 1927, members of the Genetical Society received a circular letter from the Eugenics Society appealing for their support. Signed by five eminent figures who were already members of both organisations—Fisher, plus the London embryologist Ernest W. MacBride, the Leicester physician C. J. Bond, and two biologists associated with Oxford, Julian Huxley and Alexander Carr-Saunders—the letter was intended to encourage others in the Genetical Society to follow suit and officially join the eugenical ranks. ‘[T]he importance of the heredity factor in human affairs will never be fully appreciated either by public men, or indeed by the medical profession’, the letter urged, ‘unless this and allied Societies receive the support of, and act in consultation with those biologists who have given special attention to this aspect of biology’ (MacBride et al. 1927). With Bateson no longer around to dampen enthusiasm, this latest approach yielded some success. At the next meeting of the Eugenics Society’s Council, held a month after the circular appeal, four new recruits from the Genetical Society were elected, including its surviving co-founder Edith Rebecca Saunders (Eugenics Society 1927).

Ultimately, the Eugenics Society’s tactic of aligning itself to scientific expertise failed to yield legislative results. In contrast to the United States, where testimony from geneticists helped to secure a 1927 Supreme Court ruling in support of the legality of enforced eugenic sterilisation (Lombardo 2022), and Germany, where the 1930s saw the enactment of geneticist-backed eugenic measures so extreme that they eventually opened the path to genocidal mass murder (Teicher 2020), British eugenicists emerged from the interwar period with little to show for their campaigning efforts. Their draft Sterilisation Bill was defeated in Parliament in 1931 (Hart and Carr 2015; Aylward and McGuire 2025), and thereafter the Eugenics Society’s attention steadily shifted away from agitating for eugenic policy and towards supporting demographic and population research (Soloway 1998). All the while, the organised eugenics movement in Britain endured damaging attacks from left-wing biologists, including Lancelot Hogben at the London School of Economics and, increasingly, the third President of the Genetical Society, J. B. S. Haldane (Mazumdar 1992, p 146). Our three biographical case studies start with him.

## J. B. S. Haldane

The son of an Oxford physiologist and namesake of another one, John Burdon Sanderson Haldane (1892–1964) showed early signs of becoming an Oxford physiologist himself, publishing two papers in the *Journal of Physiology* not long after his arrival at New College, Oxford in the autumn of 1911, and going on after the First World War (in which he served on the front lines) to be elected a Fellow there and to teach physiology. But though physiological research occupied him on and off throughout his long career—most dramatically so in wartime, when he subjected himself to legendary bouts of self-experimentation to better understand how to help soldiers survive gas attacks and other ordeals—he became best known for mathematically adept work in biochemistry, genetics and evolution done after he left Oxford, first at Cambridge (where he was Reader in Biochemistry from 1923 to 1932), next at the post-Bateson John Innes (where he worked part-time from 1927 to 1937), and then at University College London (UCL), where he served first as Professor of Genetics and then as the inaugural Weldon Professor of Biometry from 1933 to 1957. Over these decades, he became one of the most successful popular writers on science as well as one of Britain’s most prominent Marxist (and indeed, from the late 1930s, Communist) scientists. His final posts were in India, where he lived and worked with his wife Helen Spurway, until his death from cancer at the age of 72.

Haldane’s writings on eugenics have mainly attracted attention from historians interested in how it meshed with his leftist politics (but see also Adams 2000 and McOuat 2017). Consider, for example, two classic treatments from the mid-1980s, Diane Paul’s article ‘Eugenics and the Left’ (Paul 1984) and Daniel Kevles’ book *In the Name of Eugenics* (Kevles 1985). Paul opened with Haldane, noting that from his 1920s pre-Marxist days through the controversy over Lysenkoism in the late 1940s and beyond, he took for granted ‘the value of a socially responsible eugenics’ (Paul 1984, p 567), and that when he and others on the left criticised eugenics, they directed their objections ‘not to eugenics per se but to a eugenics which served the interests of the prevailing social order’ (Paul 1984, p 572), above all by ignoring or, worse, preserving the conditions that can mask hereditary potential at the bottom of that order. A passage on the EES in a 1924 essay on eugenics and social reform, republished in Haldane's first collection of popular essays, *Possible Worlds*, captures the core of this left-eugenical view:

The Eugenics Education Society have doubtless done good work in persuading a certain number of intelligent people that it is their duty to have more children. They have also rightly urged lessened taxation of parents of children. But many of their members have coupled this with a clamour against measures designed to ameliorate the lot of the children of the poor at the expense of the rich. It is a curious policy to combat evils due to economic inequality by perpetuating that inequality. (Haldane 1927a, p 193)

Elsewhere in the collection is a line that furnished Kevles with his title: ‘many of the deeds done in America in the name of eugenics are about as much justified by science as were the proceedings of the Inquisition by the gospels’ (Haldane 1927b, p 144). In Kevles’ portrayal, Haldane, Huxley and Hogben formed an interwar left-eugenical scientific vanguard that, for all their differences in background, temperament and—not least—attitude to eugenic officialdom (Huxley a campaigning part of it, Hogben disparaging it as the home of ‘ancestor worship, anti-Semitism, colour prejudice, anti-feminism, snobbery, and obstruction to educational progress’, Haldane between the two), helped raise the public profile of the case against eugenics in its most scientifically benighted and socially biased varieties (Kevles 1985, pp 122–128, quotation on p 123, from Hogben 1931, p 210).

Hogben, the author of *Nature and Nurture* (Hogben 1933), is now so much better remembered for an emphasis on heredity-environment interaction as undermining oversimplified genetics, pure and applied (see, e.g., Tabery 2008), that it is tempting to suppose Huxley and Haldane adopted that theme from him. Recent scholarship has revealed, however, that it featured prominently in biological teaching at Oxford back when Huxley and Haldane studied there, leaving an imprint on their future work (on Huxley, see Bernhardt-Radu 2025a). Where students at early-twentieth-century Cambridge were taught Bateson and Punnett’s deterministic Mendelism, the tone in Oxford biology was set by Mendelism’s most brilliant critic, the biometrician W. F. R. Weldon, who—far more than his fellow biometricians Galton and Karl Pearson—stressed the scope for complex interactions between germinal and environmental causes during development, along with the character variability that can result. Although Weldon died in 1906 with his book on his alternative to Mendelism unfinished, the younger men in his Oxford circle maintained his viewpoint in their own teaching, among them the comparative morphologist E. S. Goodrich (Radick 2023, pp 318–319). While Fisher at Cambridge was poring over Bateson’s *Mendel’s Principles of Heredity* (Edwards 2013), Haldane—also, formally, a mathematics student—was attending Goodrich’s biology lectures (Clark 1968, p 29), whose Weldonian take-home about visible characters was that they were never either ‘acquired’ or ‘inherited’ but always both (Bernhardt-Radu 2025b). By the time the Oxford University Eugenics Society was established in 1913, with Haldane as a founding council member, eugenics at Oxford was thought about as a heredity-environment endeavour (Aylward and Brooks in press)—and so it remained for Huxley and Haldane.

Look again, for example, at that 1924 essay of Haldane’s on eugenics and social reform, and we find, at the start of his discussion about worries over the lower classes out-reproducing the upper classes, the following: ‘We must first examine the question how far heredity rather than environment is responsible for the mental differences between the children of different social classes. The question cannot be answered on *a* *priori* grounds’ (Haldane 1927a, pp 191–192). That there was no knowing without empirical investigation how particular genotypes respond to particular environments, and so no knowing how far character differences will increase or decrease or even reverse with a change of environment, was a lesson that Haldane went on to present to both specialist and lay audiences as a wholly general one about the logic of genetics (Haldane 1936; Haldane 1938, pp 34–42; Haldane 1941, 25; Haldane 1946; Radick 2007, pp 250–251). His sense of the lesson’s importance can only have been confirmed by events around the time that he took up the Genetical Society’s presidency and moved from Cambridge to UCL (where he would eventually take up a professorship memorialising Weldon, who had taught there in the 1890s). Visiting the United States in 1932, Haldane experienced at first hand the reality of eugenics in a country where its advocates did not even try to conceal race and class prejudice, and where the Sixth International Congress of Genetics in Ithaca, New York that he attended took place at a time and place convenient for delegates coming from the Third International Eugenics Congress in New York City. (According to one recent biographer, Haldane ‘pointedly refused to attend’ the latter; Subramanian 2019, pp 192–194). Early in 1933, Adolf Hitler’s becoming Chancellor in Germany ushered in a new era there of eugenic enthusiasms and enactment, modelled on the American situation but inflected with nationalist-socialist rather than capitalist ideology (Kühl 1994). In Britain in May, the *Jewish Daily Bulletin* ran an article entitled ‘Hitlerism a Denial of a Common Civilization, Says Prof. J. B. S. Haldane’, which quoted Haldane raising the alarm among UCL students after he had spent time with Nazi intellectuals and discovered that, in their view, discussion with non-Aryans was a waste of time (Jewish Telegraphic Agency 1933, p 3).

Haldane’s identities as opponent of racialist Nazism (and supporter of German-Jewish academic refugees) and President of the Genetical Society intersected a year later, when he received a letter from one of the Society’s members, the rabbit fancier E. C. Richardson, in response to an article in *The* *Times* quoting a Nazi official, Wilhelm Kube, on the definition of a Jew as ‘one who has more than 10 percent of Jewish blood in him’ (Times 1934, p 10; Marie 2008, p 928). ‘You may perhaps remember’, wrote Richardson in late May 1934, ‘that at a Committee meeting of the G.S. held at Cambridge last autumn

I suggested that the Society might do something to combat the grossly unscientific attitude which was being adopted towards the Jews. I was not able to be present at the meeting myself, but I understand that the Committee thought, at the time, that it would be better to wait till the Nazis had committed themselves rather more deeply before taking any action. Don’t you think the passage I have underlined is fairly specific and that, since other British Scientific Societies appear to be moving in this matter, the G.S. might now take a hand? The idea that inheritance in the individual depends on ‘blood percentages’ is, as I have good reason to know, still widely prevalent; and this would seem to afford a good opportunity of stating that it is devoid of scientific foundation. We should, I submit, be acting within our province in so doing: for Rule 2 states that the Society ‘is founded to promote the advancement of Genetics’. A letter to the *Times* signed by our officers on behalf of the Society would, I am sure, meet with the approval of all members. (Richardson 1934)

Haldane explained that, although very supportive in a private capacity, he was dubious that the proposed letter fell within the Society’s functions, and so was minded to proceed with caution, since ‘more harm would be done by splitting the society than good by protesting’ (Haldane 1934). He nevertheless advised Richardson to put in a motion. The Society’s Minute Book indeed records—in an entry written by Haldane himself—that at the 1934 annual meeting, held on the afternoon of June 30^th^ at Down House, Richardson moved, and the potato geneticist Redcliffe Salaman (himself Jewish) seconded, a general proposal that ‘the Committee be empowered to express the views of the Society on any question relating to the scientific aspects of heredity raised in the press’. As Richardson observed, other societies in cognate areas—he probably had in mind the Royal Anthropological Institute, on whose behalf the anatomist Grafton Elliot Smith had sent a letter to *The* *Times* challenging the falsehoods of Nazi race biology—were raising their voices (Smith 1934). By contrast, the Genetical Society, true to its founder Bateson’s vision, still imagined itself as a place where specialists could stop their ears to the eugenical hubbub and concentrate on data, theory, and their interpretation. The proposal to publicly criticise spurious Nazi pronouncements on heredity was defeated, with five votes in favour and ten against (Genetical Society 1934).

‘Haldane mixed so many apparently contradictory traits into his persona that one word stands out in every description of him I have ever read: *enigmatic*’. So wrote Stephen Jay Gould in a fine biographical essay on Haldane, concentrating on his mid-1920s defence of chemical weapons as more humane in their effects than conventional weapons (Gould 2000, p 305, emphasis in original). Haldane’s essay quoted above on eugenics and social reform indeed begins by complaining that the ‘confused thinking’ behind the banning of mustard gas was now in danger of scuppering the benefits to democracy that well-managed eugenics could bring (Haldane 1927a, p 190). Twenty-plus years later, in response to the crisis over Lysenko, Haldane again showed willingness to make himself unpopular in the service of what he regarded as the scientifically and morally correct position. Yet when eugenics internationally was moving in directions he abhorred, at a moment when he was head of one of the world’s most distinguished societies for genetics, he proved reluctant to lead. The result was that the Genetical Society went missing in action when its authority might have made a difference for the better. Having avoided the ‘split’ Haldane so feared, the Society carried on, quietly, through the presidencies of Saunders (1936–38), the Edinburgh animal geneticist F. A. E. Crew (1938–40) and then, at the start of what became the Second World War, Fisher.

## R. A. Fisher

Born in Finchley, North London, in 1890, Ronald Aylmer Fisher arrived at Gonville and Caius College, Cambridge, in 1909 on a mathematics scholarship. He graduated in 1912 with first-class honours. In 1919, he joined the staff of Rothamsted Agricultural Station, where he developed a series of lastingly influential statistical methods. He was elected Fellow of the Royal Society in 1929, and in 1933 replaced the retiring Pearson as Galton Chair of Eugenics at UCL. Fisher’s Presidency of the Genetical Society (1940–43) coincided with his final years in London, from where he returned to Cambridge as the second Arthur Balfour Professor of Genetics, succeeding Punnett, the Genetical Society’s second President. Knighted in 1952, Fisher retired from his Cambridge chair in 1957 and later emigrated to Adelaide, South Australia. By the time of his death in 1962, he was widely considered one of the most important twentieth-century figures in the fields of statistics, genetics, and evolutionary theory (Yates and Mather 1963; Box 1978).

These biographical details are doubtless familiar to readers of *Heredity*, a journal which Fisher himself co-founded with the cytologist Cyril Dean Darlington in 1947. Also familiar, perhaps, is the controversy which surrounded Fisher in the summer of 2020, when his troubling views on race and lifelong advocacy of eugenics became the subject of a debate that eventually made its way into *Heredity*’s pages, as noted above, via an article co-authored by Walter Bodmer, himself a student of Fisher, and several other distinguished scientists. Following a summary of Fisher’s major scientific achievements, the article offered what it characterised as a ‘careful account’ of the ‘substance and nature’ of his views on eugenics and race. In concluding, the authors wrote: ‘Rather than dishonouring Fisher for his eugenic ideas, which we believe do not outweigh his enormous contributions to science and through that to humanity, however much we might not now agree with them, it is surely more important to learn from the history of the development of ideas on race and eugenics, including Fisher’s own scientific work in this area, how we might be more effective in attacking the still widely prevalent racial biases in our society’ (Bodmer et al. 2021, p 575).

Though not obvious in Bodmer et al.’s list of references, historians of science have been producing detailed scholarship on Fisher’s eugenical ideas for decades, exploring among other things his activities as a founding member of the Cambridge University Eugenics Society during his student days, his prominent role in the Eugenics Society’s campaign to legalise voluntary sterilisation of the ‘mentally deficient’, and his attempts to garner support for his own proposals for eugenic family allowances (Mazumdar 1992, pp 69–74; Barker 1989; Aylward 2021). The enduring scholarly interest in Fisher’s eugenical activities stems not from an ambition to ‘dishonour’ him or belittle his scientific achievements, but rather from the fact that he provides such a rich case-study for illuminating several important aspects of the history of eugenics in Britain.

Indeed, it is in part Fisher’s rightful status as a giant of twentieth-century science which makes him so instructive. In the shadow of WWII, and increasingly through the 1960s, it was common among scientists and historians alike to retrospectively dismiss interwar eugenics as a ‘pseudoscientific’ obsession of cranks and third-rate scientists (Paul 2016, p 643; Marks 1993). From the 1970s, though, new scholarship on Fisher by the historian Bernard Norton and sociologist Donald MacKenzie helped challenge these attitudes. That such a ‘distinguished figure’ as Fisher had harboured a lifelong devotion to eugenics demonstrated that this pernicious ideology was no fringe doctrine (Norton 1978, p 224). Rather, eugenics had evidently penetrated the very heart of the scientific establishment, occupying some of its most brilliant and influential minds.

Norton and MacKenzie went further, arguing that eugenic aims not only motivated Fisher, but also left their indelible imprint on some of his most celebrated scientific contributions. His classic paper on ‘The correlation between relatives on the supposition of Mendelian inheritance’ (Fisher 1918), for instance, must be understood as a ‘stunning contribution to eugenics’, in the way it quantitatively circumscribed the role of environment, leaving Mendelian ‘factors’ as almost sole determinants of phenotypic variation (and thus the only legitimate target for policies intended to improve population ‘quality’) (Norton 1977). Fisher’s preoccupation with the biological ‘improvement’ of humankind is perhaps most visible in *The Genetical Theory of Natural Selection* (Fisher 1930). This book opens with chapters which lay the foundations of theoretical population genetics and neo-Darwinian evolutionary theory and, following the pattern set by Bateson and Punnett, concludes with the author’s eugenic reflections, in this case, a grandiose eugenic theory of civilisational decline.

Fisher insisted in his preface that the ‘deductions respecting Man are strictly inseparable from the more general chapters’ (Fisher 1930, p x). Norton and MacKenzie agreed, arguing that the book’s eugenic moments help us to understand why, in the ‘more general chapters’, Fisher depicts evolution as ultra-selectionist, and why he devotes so much attention to issues like progress and fitness, and so little to ‘natural historical’ problems of speciation and biodiversity. This, they argue, is precisely what one ought to expect of an author drawn into evolutionary discussions principally via an interest in the selective improvement of humans (Norton 1983; MacKenzie 1981; Aylward 2025).

But one does not need to go all in for social constructivism to see that eugenics was central, rather than incidental, to Fisher’s science. Indeed, Fisher’s case shows us that, for some in the early twentieth century, eugenics could provide a route—albeit an unorthodox and precarious one—into a scientific career. After graduating from Cambridge, Fisher endured a brief spell working for the Mercantile and General Investment Company. Though professionally unfulfilling, his employment in London at least enabled him to spend ample time at the offices of the EES, then based at Kingsway House in Holborn, where he built a close relationship with President Major Leonard Darwin, a son of his hero Charles Darwin (Serpente 2016). With the outbreak of war, Fisher, who was barred from active service on account of his poor eyesight, left the city for a school teaching position. Eager to hold onto this promising young eugenicist, Darwin made room in the EES’s tight budget to cover Fisher’s weekly train fare into London plus an annual salary of £100 in exchange for ‘about one quarter of your working time, this time to be spent on the Society’s business or, in default of that, on eugenic investigation’ (Box 1978, p 51).

The arrangement kept Fisher plugged into the scientific culture of the capital throughout a critical period in his early career. He would write and publish several important early papers, including his famous ‘correlations’ article (Fisher 1918), from the comfort of the EES offices, where he enjoyed unfettered access to the Society’s well-stocked library. Here, he continued his self-education in the new science of genetics. At Cambridge, he had devoured Mendelian texts by Bateson, Punnett and others (Edwards 2013), digesting their core lessons in addresses to fellow undergraduate members of the Cambridge Eugenics Society (Fisher 1911). Now, Fisher spent hours among the EES library stacks, cataloguing new accessions and reviewing dozens of genetics books and streams of periodical literature for the Society’s journal, *The Eugenics Review*. Even once he secured a full-time post as statistician at Rothamsted Experimental Station in 1919, Fisher committed to continuing on a voluntary basis this previously paid duty of penning summaries of new genetic literature. The eugenicist rank and file knew too little genetics, and it was hurting their cause. Fisher was determined to change this.

Fisher’s growing status through the 1920s as a leading expert in both statistics and genetics was a major boon to the Eugenics Society. Increasingly, they leaned on his authority, especially during their energetic but ultimately unsuccessful campaign to legalise voluntary sterilisation of ‘mental defectives’. In a 1924 article in *The Eugenics Review*, Fisher calculated that, assuming mental deficiency is inherited as a Mendelian recessive, based on his reading of Punnett (1917), a policy of sterilising manifest defectives could be expected to reduce the affliction’s incidence by 17.4% in one generation, and over 40% after three generations (Fisher 1924). As the Society stepped up its propaganda efforts, it printed and distributed thousands of copies of Fisher’s paper, and prominently cited his calculations in publicity materials (Aylward and McGuire 2025). Despite their efforts, the Society’s sterilisation bill was defeated by 167 votes against versus 89 in favour when eventually introduced at parliament in July 1931 (Hart and Carr 2015).

A decade later, in 1941, Fisher resigned from the Eugenics Society’s Council (Fisher 1941). That he did so one year into his three-year term as President of the Genetical Society appears strange, given his longstanding commitment to strengthening ties between the two communities. But he had by this time already retreated significantly from the Eugenics Society, which he felt had gradually lost its way since the parliamentary defeat of its sterilisation bill, consistently sidelining genetics professionals and other scientific experts, while bending increasingly to the environmentalist and egalitarian critiques of left-leaning ‘reform eugenicists’ (Aylward 2021; Mazumdar 1992; Soloway 1998). By the early 1940s, the new science of human genetics was going from strength to strength, thanks in no small part to Fisher’s own efforts. But the Eugenics Society—the body ostensibly tasked with harnessing this new knowledge of human inheritance towards eugenically-minded policy—seemed to him ill-prepared, even unwilling, to take advantage (Fisher 1935). The science of genetics and the political project of eugenics were diverging, Fisher thought, to the detriment of both.

Fisher published little on eugenics after the Second World War, yet this hardly reflected a softening of his views. Indeed, much of the recent Fisher controversy has centred on his post-war activities, especially his decision to support the rehabilitation of Nazi-affiliated human geneticist Otmar Freiherr von Verschuer. In a 1948 letter of reference, Fisher attested to the ‘unquestioned value’ of von Verschuer’s pre-war research, while affirming that, had he been in von Verschuer’s position, he too would have lent his support to the Nazis who, Fisher was sure, had ‘sincerely wished to benefit the German racial stock, especially by the elimination of manifest defectives, such as those deficient mentally’ (Weiss 2010, p 745). Fisher’s backing would have carried weight, not only because of his scientific pre-eminence, but also due to his status as Cambridge professor of genetics, and recent president of the Genetical Society (1940–43). These credentials also gave extra prominence to Fisher’s criticisms of the 1952 UNESCO statement on ‘The Race Concept’, notably his insistence that ‘available scientific knowledge provides a firm basis for believing that the groups of mankind differ in their innate capacity for intellectual and emotional development’, due to the fact that ‘such groups do differ undoubtedly in a very large number of their genes’ (UNESCO 1952 p 56; Brattain 2007). Though by now largely estranged from the formal eugenics movement, Fisher continued through private and public pronouncements to defend its aims and its purported scientific foundations.

In some ways, Fisher was a typical British eugenicist—a member of the professional middle-class, highly educated, alarmed by apparently stark differences in fertility between social classes (MacKenzie 1976). In others, he was atypical, and instructively so. He was recognised, in his time as in ours, as an exceptional scientist who made fundamental and lasting contributions to several fields. This was useful to the Eugenics Society, who mobilised his expertise very deliberately. It has also been useful to generations of historians seeking to dispel the myth that eugenics was the sole preserve of a pseudoscientific fringe. Fisher also sits uneasily against the so-called mainline-reform axis, which structures much of the historical scholarship on British eugenics (Kevles 1985). His scientific sophistication and statistical outlook made him an astute critic of the older ‘mainliners’ (Mazumdar 1992, ch 3), while his political conservatism as well as his dogged elevation of heredity over environment as the principal cause of phenotypic variation left him at odds with the ‘reform’ eugenicists—Huxley, Haldane, Hogben, among others—who would exert such influence on the movement’s direction (or as Fisher saw it, misdirection) from the 1930s. Perhaps no ‘reform’ eugenicist had a greater impact than the man who inherited Fisher’s Galton Chair at UCL, Lionel Penrose.

## L. S. Penrose

Lionel Sharples Penrose was born in 1898 to a family of Quakers (Harris 1974; Watt 1998). In 1919, after service in the First World War in the Friends Ambulance Unit, he joined St John’s College in Cambridge, where he took his degree in the Moral Sciences Tripos (Harris 1974). He became interested in abnormal psychology in 1921 after going to Vienna to study under Sigmund Freud. Upon his return to the UK, he started a medical degree in Cambridge alongside clinical work at London’s St Thomas’s Hospital, convinced that medical training was necessary for scientists studying mental disorders. In 1931, he was appointed Medical Research Officer at the Royal Eastern Counties Institution at Colchester, a post which culminated in the Colchester Survey (Penrose 1938), and which defined his career. He then moved to Ontario, Canada, in 1938, where he worked as Director of Psychiatric Research at the London Psychiatric Hospital. He returned to the UK in 1945 to become the Galton Chair of Eugenics at UCL, succeeding Fisher. After retiring from UCL in 1965, he became Director of the Harperbury Hospital’s Kennedy-Galton Centre (Harris 1974). He died in London in 1972.

Considered alongside the entries into human genetics of Haldane and Fisher, Penrose’s was singular both in its timing—the mid-to-late 1920s—and in its scientific character—as a next step from Freudian psychiatry. It was in wartime France that Penrose first heard a lecture on Freud’s theory of dreams, which impressed him as providing a ‘reasonable explanation’ of what he described as ‘the apparently disordered sequence of ideas in the nocturnal theatre with an audience of one’ (Penrose 1998; Kevles 1985, p 152). This experience led him to enrol in Cambridge’s Moral Sciences Tripos, studying mathematics, logic, psychology and philosophy, though psychology remained his main interest. Disappointed by the lack of Freudian studies in the Tripos, however, he left for Vienna, where he stayed until 1924 to have an opportunity to study with Freud. Between 1925 and 1931, Penrose published on various aspects of Freudian theories, including inhibition, suggestion, negation and Freud’s theory of instincts, but also on psychosis and schizophrenia, indicating his growing interest in ‘abnormal psychology and mental disorder’ (Harris 1973, p 552). Penrose’s return to Cambridge for medical studies, and subsequent doctorate on schizophrenia at Cardiff City Mental Hospital ensured his Freud-inspired interest in the ‘abnormal mind’ was now coupled with cutting-edge medical knowledge (Kevles 1985, p 154; Harris 1973, p 522).

The subsequent stage of Penrose’s career was characterised by scientific opposition to eugenics. As we have seen, by the 1930s, with the complexities of inheritance and development becoming ever more clear, prominent biologists were beginning to challenge the hereditarian assumptions of traditional eugenics—assumptions which appeared to them increasingly ‘slipshod in method, evidence, and reasoning’ (Kevles 1985, p 122). In Britain, Hogben’s 1931 book *Genetic Principles in Medicine and Social Science* launched a stinging attack upon the scientific arm of the eugenics movement, including lengthy critiques of the work of Fisher, which Hogben dismissed as simplistic in its genetic determinism (Tabery 2008; Tabery 2014). Hogben was, like Penrose (whose assistance Hogben acknowledged in *Genetic Principles*), a Quaker, a pacifist, and a man of the Left. The two men bonded, corresponding regularly through the 1930s, 40s and 50s, and encouraging and praising one another’s work (Penrose Papers 1931–1933, PENROSE 3/8/24).

While Hogben led the charge with an influential environmentalist challenge to eugenics, Penrose gladly followed, citing his friend liberally as he did so (Penrose 1932a; 1932b). A good example of Penrose’s critical stance in this period is his 1934 Buckston Browne Prize essay on the influence of heredity on disease. The essay argued for the centrality of environmental effects in the development and expression of various diseases, while at the same time remarking on the question of who possessed the professional authority to adjudicate the relative importance of environment or heredity in disease aetiology. Penrose was clear: this was the job of the medically-trained physician, and *not* the detached statistician—a direct attack upon the meddling of eugenicists, and specifically Fisher and Pearson, whose work he singled out for special criticism (Penrose 1934, p 14; Mazumdar 1992, p 221).

Take tuberculosis. This disease, Penrose explained, would not exist in populations genetically susceptible to it if it were not already present in the environment. *Prevention* through environmental reform, therefore, represents by far the best, most humane and most efficient way to eradicate diseases, compared with the Eugenics Society’s favoured alternative of sterilisation (Penrose 1934, pp 73–74). More generally, Penrose challenged the idea that any ‘expert’ group might decide which traits are eugenically ‘desirable’ and which are not, given that in some environments ‘undesirable’ traits are actually beneficial; ‘What is pleasant for the individual, is not necessarily good for the perfection of the “race”’ (Penrose 1934, p 76). The essay is littered with references to the work of Hogben, who, alongside Haldane, Penrose credits with laying the foundations of ‘a science of human genetics which must be considered an important branch of medical study’ (Penrose 1934, p 8; Mazumdar 1992). Eugenics, by contrast, was firmly *un*scientific, founded on prejudice and dealing in ‘hearsay evidence’ (Penrose 1934, p 71).

At a moment, then, when the central role of eugenics in Fisher’s science was visible as never before, Penrose made it his mission to discredit what he saw as ideology masquerading as science. For Penrose, the practical aim of genetics must be the treatment and prevention of disease (Penrose Papers 1969, PENROSE/2/45/6/5), rather than the creation of an ‘elite group’. Although it is hard to say exactly how far Penrose’s humanitarian streak can be attributed solely to his Quaker upbringing, descriptions from those who worked with him on Down syndrome evoke a recognisably Quaker sensibility. He was known to play with his patients to understand their condition, approaching them ‘as a personal friend’ (Smith 1998), creating an ‘affinity and harmony’ which resulted in a ‘festive atmosphere’ whenever he visited them (Fraser 1998). He listened with ‘kindness and understanding’, believing them to have ‘a particular temperament … a secret source of joy’ (Harris 1973; Kevles 1985; Fraser 1998, p 42). Contrast this with the reliance of eugenicists on large and impersonal statistical surveys, in which individual ‘defectives’ were routinely reduced to mere data points (Aylward and McGuire 2025).

Penrose’s challenges to eugenics continued with his Colchester Survey, published in 1938. There, Penrose concluded that so-called ‘mental deficiency’, presumed by traditional eugenicists to be straightforwardly inherited, in fact arose from a complex mix of genetic and environmental causes (Harris 1973, p 526). In later work on phenylketonuria (PKU), Penrose identified interactions between heredity and environment for a Mendelian-recessive condition (Penrose and Quastel 1937; Wright 2001, p 172; Penrose 1949b), rejecting a view of mental disabilities as the exclusive product of ‘arrested brain development or brain damage [that] could be passed down to later generations’ (Penrose and Quastel 1937; Wright 2001, p 172; Penrose 1949b). The staunchly hereditarian understanding, so favoured by eugenicists, of the causes of mental disability was gradually being overthrown (Barkan 1992, p 201). Penrose never became closely involved in the activities of the Eugenics Society, though he did occasionally accept invitations to present papers at their meetings. In a paper read before the Society in 1949, Penrose quipped that the apparently excessive fertility of what was commonly referred to as the ‘social problem group’ revealed them to be biologically fitter than their socioeconomic ‘superiors’. If fitness amounts to the ability to survive and *reproduce*, then surely ‘the superiority of the infertile intellectuals is illusory’ (Penrose 1949a, p 23). Penrose made a regular habit of ridiculing eugenic ideas by turning them on their heads. By the early 1950s, leaders within the Society judged that Penrose’s ‘pronouncements now constitute just about our most serious problem’ (Blacker 1951).

It was somewhat ironic for such a vocal critic of eugenics to be serving as UCL’s third Galton Professor of Eugenics. Though his predecessor, Fisher, had disapproved of the appointment, Penrose had been the preferred choice of Haldane, UCL’s Weldon Professor of Biometry. Writing in *Nature* in February 1945, Haldane welcomed his new colleague, highlighting Penrose’s ability to combine the statistical tools of his predecessors with ‘clinical and psychological methods’. Whereas the previous two occupants, Fisher and Pearson, had arrived at eugenics from ‘mathematics via statistics’, Penrose came ‘from psychology via medicine’. This medical expertise meant that, with Penrose as Galton Chair, Haldane expected that ‘the eugenic movement will become a good deal more concrete’ (Haldane 1945).

In his time as Galton Professor, Penrose indeed oversaw much ‘concrete’ work, especially on medical aspects of human genetics (Penrose 1949a). But it was clear from the outset that he did not view this research as contributing to the eugenic project. Indeed, the very word eugenics troubled and embarrassed him. From early on, he pointedly avoided writing his correspondence on notepaper with a ‘Department of Eugenics’ letterhead (Robson 1998). Later, he sought to formally expunge the word from his department, beginning with the in-house journal, *Annals of Eugenics*. Founded by Karl Pearson in 1925 and traditionally under the editorship of the Galton Chair, *Annals* originally carried the subtitle *A Journal for the Scientific Study of Racial Problems*. Under Fisher, it became *A Journal devoted to the Genetic Study of Human Populations*. This was edited further by Penrose to *A Journal of Human Genetics* before, in 1954, he moved to change the journal’s title altogether to *Annals of Human Genetics*. An editorial note in the first issue under the new moniker explained: ‘From the outset, the journal contained many papers dealing with heredity and, in recent years, has consisted almost exclusively of contributions to the science of human genetics. It seems logical to recognise this trend by the alteration of the title’ (Penrose 1954a). Penrose took the opportunity of discontinuing the journal’s old title to emphasise that the *scientific* study of human heredity had long left eugenics behind.

When Penrose broached the matter of changing the journal’s title in 1953, his colleague Haldane was supportive, remarking ‘I have no possible objection’ (Haldane Papers (multiple dates), HALDANE/5/2/4/19, f.33). Though he did not actively resist change, it was in fact Haldane’s retirement in 1957 which paved the way for further reform at UCL. With Haldane’s departure, Penrose was elevated to Head of what had by now evolved into the Department of Eugenics, Biometry and Genetics, and could initiate the process of its renaming (Senate Meetings multiple dates, UoL ST/2/2; AR 485 n.d.). In this, Penrose faced various administrative and legal obstacles to altering Galton’s 1911 bequest, with administrators expressing horror at all levels for the administrative, legal and financial complexities and ramifications. He did, though, have the strong support of UCL’s Provost Ifor Evans, and with perseverance, he eventually succeeded. By 1963, almost twenty years after arriving at UCL, Penrose found himself head of the Department of Human Genetics and Biometry (Senate Meetings multiple dates, UoL ST/2/2; AR 485 n.d.; Kiladi 2021; Kiladi 2025). Under his direction, the Galton Laboratory had gradually shifted its focus from pedigrees and statistical surveys to experimental medical and biological studies, emerging as a ‘mecca for aspiring human geneticists from England, the Empire, the United States and the Continent’ (Kevles 1985, p 222). Finally, in Penrose’s view, the department’s name truly reflected the nature of its research.

Between them, then, Penrose’s Quaker upbringing, his training in biologically attuned but unreductive psychoanalysis, and his coming to genetics as a medical researcher in the late 1920s prepared him to contribute to the work of scientific critics who attacked mainstream eugenics from the early 1930s and throughout the mid-century. On a personal level, Penrose always observed a sharp distinction between what he saw as the ideology of eugenics versus the science of genetics. More, perhaps than anyone else in the British context, his activities aimed to make such a distinction widely felt. Accordingly, he placed extreme pressure on the hereditarian understandings of mental (dis)ability, which had long sustained calls for eugenic action through segregation and sterilisation. In practical terms, the reforms he led at UCL deprived eugenics of its securest foothold in the UK academy.

## Eugenics, genetics, and scientific critique

The tenures of our past Presidents span a period across which the status and fortunes of both eugenics and genetics were significantly transformed. When Haldane took over from Punnett, it remained common even among its critics to conceive of eugenics as an applied science, one which sought to put into practice the findings of what was viewed as pure genetics. By the end of Penrose’s reign in the late 1950s, genetics was thriving, its rising disciplinary status reflected in new departments, chairs and journals. Eugenics, meanwhile, was on the wane, dismissed by many as outmoded pseudoscience.

One sometimes encounters the view that the growth of genetics as a science directly precipitated eugenics’ mid-century demise (Ludmerer 1969). According to this view, as genetics matured and discoveries of the complexities of heredity and the intricacies of development progressed, eugenic visions of populational improvement via selection came to seem increasingly implausible. By the outbreak of the Second World War, eugenics was already a movement in decline, its scientific foundations (if it ever had any) substantially diminished. This comforting narrative emphasises the power of sophisticated science to deliver us from prejudice, while drawing a sharp line between modern genetic science and the discarded eugenic pseudoscience of times past. Recent historical scholarship shifts this story.

Rather than a dramatic post-1945 fall from grace, scholars nowadays tend to emphasise the continuity of eugenics through the later twentieth century and even into the present (Bashford 2010). Following the Second World War, dedicated eugenics organisations marched on (Mazumdar 1992), and sterilisation programmes in the US, Canada and elsewhere continued unabated (Wilson 2017). In the scientific arena, eugenic ideas continued to inform mainstream research, while eugenic terminology survives today in the pages of reputable journals (Kampourakis and Peterson 2023). Clearly, the accumulation of scientific discoveries in our understanding of heredity did not stop eugenics in its tracks. To be sure, certain genetic advances did sometimes lead to questioning of eugenic aims and arguments. At other times, however, they did not, and eugenics advocates often attempted to accommodate, or make a virtue of, new and more sophisticated knowledge about heredity (Mazumdar 2002; Paul and Spencer 2001). This section uses past Presidents of the Genetics Society to illustrate how prominent geneticists deployed increasingly complex genetic theory and empirical research to support, as well as to refute, eugenic proposals, with examples from the arena of eugenic attempts to eliminate ‘mental defect’. Haldane, and especially Fisher and Penrose, made significant contributions to this discourse, infusing population and quantitative genetics into their arguments, but to widely opposing ends.

From a genetics standpoint, the complex and troubling history of efforts to solve the ‘problem’ of mental deficiency is a cautionary tale. Take the category of ‘mental deficiency’ itself, sometimes subdivided into ‘idiots, imbeciles, feebleminded persons, and moral defectives’ (Penrose 1949b, p 18). These terms, now discarded as ill-informed and offensive, were rife in eugenics discourse through the first half of the twentieth century. Although clearly social categories, they were routinely portrayed as strongly, if not exclusively, genetic. American psychologist Henry Herbert Goddard (1910) published multiple pedigrees displaying purported evidence of recessive Mendelian heredity for feeblemindedness. He made the same claim in his wildly popular book *The Kallikak Family: A Study in the Heredity of Feeblemindedness* (Goddard 1912). He followed this with a technical book, *Feeble-Mindedness* (Goddard 1914), in which he presented claims of genetic and environmental causes of feeblemindedness, but again with a focus on recessive Mendelian heredity.

Goddard’s research would later be shown to be strewn with confirmation bias, embellishment and fabrication (Smith and Wehmeyer 2012; Barker 1989; Smith 1985). But this did not prevent it from exerting significant influence among his peers (and, later, on Nazi propaganda). Noted Harvard geneticist Edward East (1917), for instance, cited Goddard (without a reference) to assert that ‘feeblemindedness is transmitted as a Mendelian recessive’ (p 215). East’s article would, in turn, inspire future Genetical Society President Reginald Punnett. In consultation with G. H. Hardy, Punnett calculated that the purported recessive nature of feeblemindedness meant a programme of sterilisation or segregation of individuals diagnosed as feebleminded was doomed to failure, by showing mathematically how the effectiveness of selection against a recessive allele declines with repeated generations of selection. If phenotypic frequency of feeblemindedness was 3 per 1000, as estimated by East (1917), ‘it would require something over 250 generations, or about 8000 years before the proportion was reduced to 1 in 100,000’ (Punnett 1917, p 465).

This may seem like a straightforward case of an expert applying genetic theory to undermine eugenics. But this was not Punnett’s intention. A devout eugenicist, Punnett did not conclude that the project of racial improvement was hopeless, but merely that ‘if that most desirable goal of a world rid of the feebleminded is to be reached in a reasonable time some method other than that of elimination of the feebleminded themselves must eventually be found’ (p 465). He proposed identifying heterozygous carriers and restricting their reproduction based on his and East’s speculation that feeblemindedness might not be fully recessive. Nevertheless, some of the movement’s critics mobilised his calculations as evidence of the futility of eugenic policies (Paul and Spencer 2001, p 107).

In 1924, Fisher entered the fray. His article on the ‘elimination of mental defect’, discussed above, was a direct rebuttal of Punnett’s ‘inadvertently supplied material for anti-eugenic propaganda’ (Fisher 1924, p 114). He argued mathematically that, even under Punnett’s assumptions, eugenic progress was possible within just one generation, with continued progress in the second and third generations. He then questioned Punnett’s genetic assumptions. Conceding that ‘there is a considerable body of pedigree evidence of a single mendelian factor’, he argued further that ‘there are doubtless many mendelian factors of sufficient importance to decide the fate of borderline cases; possibly there are several factors each capable alone of producing feeblemindedness’ (p 115). The genetic basis of feeblemindedness, he stressed, is likely to be multifactorial and in other ways more complicated than Punnett and others assumed. Indeed, the prospects of eliminating mental defect looked much rosier to Fisher, once he corrected what he asserted to be a faulty assumption—namely, Punnett’s neglect of assortative mating. Punnett had assumed random mating, but according to Fisher (1924), ‘Mating is very largely controlled by social class, and the feebleminded undoubtedly gravitate to the lowest social stratum’ (p 115). Relying on Goddard’s (1914) data, supplied to him by Leonard Darwin, Fisher concluded that ‘The concentration of the defect in a limited number of strains … renders the probable rate of progress as great as any to be expected in the field of diseases capable of remedial control’ (p 116). Presented differently than Punnett’s, Fisher’s calculations were more eugenically promising (Aylward and McGuire 2025). The inclusion of greater nuance and complexity actually boosted, rather than harmed, the prospects of eugenics.

Much of the back-and-forth of the 1910s and 20s relied on the largely unquestioned assumption that mental deficiency owed primarily, if not exclusively, to an individual’s genotype. Penrose’s writings from the 1930s onward represent a crucial shift away from hereditarian approaches toward quantitative-genetic interpretations with substantial nongenetic components contributing to ‘mental defect’, including ‘feeblemindedness’, substantially influenced by his Colchester Study (Penrose 1938). He laid out these interpretations in his 1949 book *The Biology of Mental Defect*, with Haldane underscoring their importance in a preface:

The demonstration that some cases of mental abnormality were largely genetically determined led to exaggerated hope of eugenical improvement. The demonstration that others could be improved by hormones, psychotherapy, or special teaching methods led to equally exaggerated hopes of another kind. Both schools of thought underestimated the immense complexity of the problem. This was first concretely shown in Penrose’s now classical report (Colchester Survey) to the Medical Research Council in 1938, in which he demonstrated that mental defect could be due to a vast variety of different causes. (Haldane in Penrose 1949b, p v)

The development of quantitative genetics, due in large part to the theoretical work of Haldane and Fisher dramatically transformed plant and animal breeding in the 1930s. Penrose’s incorporation of quantitative-genetic theory into human genetics came shortly thereafter, evident in *The Biology of Mental Defect* (Penrose 1949b and subsequent editions) and several of his other publications (Penrose 1948, 1950, 1954b). Specifically, Penrose applied partitioning of genotypic variance into additive and non-additive components (such as dominance deviation) to address observed parent-offspring correlation, as originally proposed by Fisher (1918), to show that observations of variation for ‘mental defect’ were inconsistent with an overriding genetic influence. For instance, he noted that parent-offspring correlations provided evidence against a strong additive genotypic component, and he addressed but downplayed the possibility that deviations from additivity were due to dominance:

The observed values [of parent-child and sib-sib pair correlations] close to 0.5 are, in fact, nowhere near what would be expected on the multiple additive factor hypothesis. There is plenty of room for the assumption of other sources of variation.

Dominance and recessivity of the genetical factors will explain the discrepancy to some extent because they reduce correlation values. It is known that there are numerous rare recessive genes causing defects of intelligence, but it is generally believed that most of the common genes responsible for normal variations are additive. If so, the magnitudes of the correlations obtained are not as conclusive evidence of the strength of genetical forces in determining intelligence level as has often been supposed. (Penrose 1950, pp 130–131)

Moreover, he showed that parent-offspring coefficients may be inflated due to the confounding of genetic and non-genetic environmental variation, directly contradicting previous hereditarian claims:

A certain amount of likeness of sibs must be attributed to similar surroundings, in the family and in the home. The real coefficients—which measure purely genetical effects—are possibly all somewhat lower than those observed. (Penrose 1949b, p 118)

In the end, he concluded that eugenic efforts were certain to be ineffective given the lack of evidence that an additive-genetic model could explain observed variation and parent-offspring correlations, with the implication that environmental variation plays a much greater role than previously assumed. Hence, it followed for Penrose that a ‘slight change in the direction of a more favourable environment during one generation could easily swamp any effect due to changes in gene frequency caused by differential fertility’ (Penrose 1949b, p 121).

Ironically, given his own ardent eugenic support, Fisher’s quantitative tools, when trained by Penrose upon the concrete problem of ‘mental defect’, yielded powerful critiques of eugenics. Fisher had first developed his methods for partitioning variance in the context of illustrating the compatibility of Mendelian and biometrical understandings of heredity, while simultaneously in demonstrating a very circumscribed role of environmental differences in accounting for phenotypic variation (Fisher 1918). This was, as Fisher (1919) pointed out at the time, a boon to eugenics, supporting as it did the notion that lasting improvements in population ‘quality’ could only be achieved through policies influencing the relative reproduction of different ‘stocks’ (as opposed to reforms aimed at improving social environment). Decades later, in Penrose’s hands, the same methods allowed him to argue for a *greater* role for environmental variation in influencing mental deficiency than previously assumed; a conclusion which, in turn, spoke strongly against the presumed efficacy of any programme of eugenic selection. Where Fisher had earlier exposed the unsound science of Punnett’s ‘anti-eugenic propaganda’, Penrose now turned the tables once more. Statistical tools designed by Fisher originally to bolster eugenics were re-deployed decades later to undermine it. We like to think that advancements in genetic science have delivered us from the errors and harms of a eugenic past. Historically, though, scientific advances did not always prove damaging to the eugenicists’ cause. Indeed, new and more sophisticated genetic knowledge and methods were used to promote eugenics as well as to discredit it.

## Conclusion

Our chosen past Presidents of the Genetics Society demonstrate some of the diversity in the ways leading geneticists interacted with the eugenics movement during the height of its influence. Fisher, for one, was a staunch supporter who embraced eugenics as an undergraduate student and remained an adherent through the end of his life. But even he grew disillusioned with the Eugenics Society, formally withdrawing from its Council in 1941, in part because ‘its present directors are strongly entrenched and appear almost impervious to scientific advice’ (Fisher 1938 as quoted in Bodmer et al. 2021). Haldane also caught the eugenics bug at university. Less active than Fisher within the Eugenics Society, and more critical of the classist excesses of eugenic ‘mainliners’, Haldane nevertheless remained open throughout his life to the scientifically guided control of human evolution.

As a critic, Penrose went much further than Haldane, rejecting outright the eugenic project of selective population improvement. His published critiques of eugenical ideas carried authoritative weight, but just as important were his efforts to rebrand UCL’s Galton Laboratory and its in-house journal. In doing so, he stripped eugenics of its most visible and respectable institutional resources. Penrose recognised that defeating eugenics was not just a scientific problem, but a political one too.

This remains true today. If the history of the interactions between eugenics and genetics tells us anything, it is that political movements may continually adapt in response to a constantly changing scientific landscape. The flexibility of eugenic and other political ideologies means they remain stubbornly persistent, even in the face of robust scientific critique (Spencer and Paul 1998; Allen 2011; Nobles et al. 2022; Fonseca et al. 2023; Bird et al. 2024). Still today, sophisticated and well-intentioned genetic science can be weaponised towards harmful and hateful ends, as we tragically witnessed in the case of the Buffalo mass shooting in 2022, wherein the perpetrator produced a lengthy manifesto detailing egregious misreadings of professional genetics research, which he took as justifying his racist killings (Carlson et al. 2022).

What responsibility do professional genetics bodies have when it comes to combatting harmful (mis)appropriations of genetics research? What, practically, can they do? These questions are not new—on the contrary, they have occupied the Genetics Society throughout its long history. Historically, even as individual members (and Presidents) threw themselves into public debate on the application of genetic knowledge to humans, the Genetical Society as an organisation pursued neutrality. True, it largely maintained a cautious distance from the organised eugenics movement in Britain, while on the other hand, its leadership declined to condemn the rise of Nazi race science. At the time, some within the Genetical Society considered this a mistake. In September 1930, prompted by the Eugenics Society’s latest proposals for legalising sterilisation of mental defectives, Redcliffe Salaman (who would later support E. C. Richardson’s unsuccessful motion to the Genetical Society’s Committee, discussed above) wrote indignantly to the editor of *The Times*:

Sir,—Like many other students of the science of heredity, I have been content to devote my time and energies to the theoretical and experimental side, leaving such practical applications as may be from time to time permissible in the hands of others presumably better qualified. Indeed, the school of pure genetics in this country has held itself rather sternly aloof from such bodies as the Eugenics Society, which sets out primarily to deal with genetics as an applied science. Personally, I feel that this has not been a wise procedure; retribution has overtaken us. (Salaman 1930)

Salaman feared that the Genetical Society’s policy of public silence on political matters was allowing eugenics to proceed unchecked, and for eugenicists to speak as though the genetics profession was united behind them. Almost a century later, the landscape is changing as scientific societies, the Genetics Society among them, are confronting political and social issues in their publications, meetings and public-outreach efforts. Nonetheless, concerns similar to Salaman’s remain.

When Bateson cautioned against close collaboration with the Eugenics Society, he did so not in the interest of condemning eugenics, but rather of protecting genetics—the science he was busily building. Later, fears of creating rifts within a small and precarious scientific community dissuaded Haldane from steering the Genetical Society toward societal and political engagement. Now, almost a century later, the status of genetics as a scientific field, and of the Genetics Society as one of its leading professional organisations, is much securer. What seemed a risk too far for the Society under Haldane—namely, using its platform to combat hateful misuse of the science of heredity—is now not only a possibility, but a responsibility.
